# A Swiss army knife for targeting receptors

**DOI:** 10.7554/eLife.37413

**Published:** 2018-05-24

**Authors:** Johansen B Amin, Lonnie P Wollmuth

**Affiliations:** 1 Program in Cellular and Molecular BiologyStony Brook UniversityStony BrookUnited States; 2 Medical Scientist Training Program (MSTP)Stony Brook UniversityStony BrookUnited States; 3 Center for Nervous Systems DisordersStony Brook UniversityStony BrookUnited States; 4 Department of Neurobiology and BehaviorStony Brook UniversityStony BrookUnited States; 5 Department of Biochemistry and Cell BiologyStony Brook UniversityStony BrookUnited States

**Keywords:** NMDA receptor, allosteric modulator, pharmacology, neurotransmitter, glutamate, None

## Abstract

A compound can change the activity of NMDA receptors in some regions of a synapse without affecting those in other regions.

**Related research article** Perszyk RE, Katzman BM, Kusumoto H, Kell S, Epplin MP, Tahirovic YA, Moore RL, Menaldino D, Burger P, Liotta DC, Traynelis SF. 2018. An NMDAR positive and negative allosteric modulator series share a binding site and are interconverted by methyl groups. *eLife* **7**:e34711. doi: 10.7554/eLife.34711

Most medicines are fairly blunt devices, which means that clinicians are often unable to account for the uniqueness of a patient when treating a specific ailment. A drug that could be modified to achieve different aims – like a Swiss army knife – would help to address this problem.

The cells of the nervous system talk to each other at structures called synapses, where the electrical signal in the first neuron is converted into a chemical signal that is carried to the second neuron by molecules called neurotransmitters. When the neurotransmitters reach the second neuron they interact with receptor proteins that are directly coupled to ion channels. Glutamate is the most prominent neurotransmitter in the brain, and glutamate-gated NMDA receptors are involved in almost every process in the brain: whether you are thinking about something or doing something, NMDA receptors are involved.

With the good, however, comes the bad: faulty NMDA receptor activity contributes to numerous neurological and psychiatric disorders. And although the central role of NMDA receptors in brain disorders has long been known, finding treatments that target such receptors has proven challenging (Kalia et al., 2008). The problem starts with the ubiquitous distribution of NMDA receptors. A drug that blocks all NMDA receptors (a so-called broad-spectrum inhibitor) will have many detrimental off-target effects, so drugs that only act on the receptors involved in specific diseases are needed.

How does one target something as complex as an NMDA receptor, which contains four subunits? It helps to know that two of these are always so-called GluN1 subunits, and that the other two subunits can be selected from a pool of four GluN2 subunits (Figure 1). These last two subunits confer unique physiological and pharmacological properties on the NMDA receptors, which can therefore behave differently, depending on the region of the brain or the stage of development (Glasgow et al., 2015; Paoletti et al., 2013). To date, researchers have focused on NMDA receptors in which the two GluN2 subunits are the same, but most of the NMDA receptors in the brain are thought to contain two different GluN2 subunits (Tovar et al., 2013). Targeting the different subunits of NMDA receptors has resulted in some progress, but additional aspects are needed to encompass the full complexity of NMDARs (Ogden and Traynelis, 2011; Hackos and Hanson, 2017).

**Figure 1.**
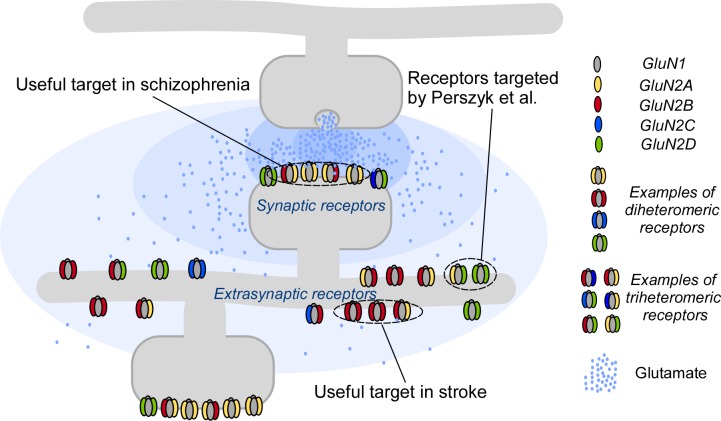
Targeting NMDA receptors. A neuron (gray; top) communicates with a second neuron (gray; bottom) by releasing molecules called neurotransmitters into the synapse between the two neurons; the neurotransmitters then bind to and activate receptor proteins on the second neuron. The neurotransmitter glutamate (blue dots) and the NMDA receptors (colored shapes) it binds to, are essential for most processes in the brain. Many disorders, including schizophrenia and stroke, are associated with faulty activity of the receptors. NMDA receptors contain two GluN1 subunits (gray ovals) and two GluN2 subunits (colored ovals). Perzsyk et al. discovered a chemical compound that can bind to receptors outside the synapse (extrasynaptic receptors), where glutamate levels are low (light blue shaded areas). Synaptic receptors, where glutamate levels are high (dark blue shaded areas), are an ideal target for treatment of schizophrenia.

Now, in eLife, Stephen Traynelis of Emory University and colleagues – including Riley Perszyk as first author – report how specific compounds can modulate NMDA receptors in unique ways (Perszyk et al., 2018). The researchers identified different chemical compounds to tackle another aspect of NMDA receptor diversity: their distribution on the neuron.

NMDA receptors can be synaptic (that is, they reside inside the synapse) or extrasynaptic (outside the synapse). These different pools of receptors behave in distinct ways. For instance, synaptic NMDA receptors activate pathways that are necessary for the survival of cells, whereas extrasynaptic NMDA receptors can induce pathways that lead to the death of cells (Papadia and Hardingham, 2007). Indeed, extrasynaptic NMDA receptors have been identified as the primary culprit responsible for cell death in stroke and Huntington’s disease (Parsons and Raymond, 2014). However, we have lacked ways to target extrasynaptic NMDA receptors.

Perszyk et al. tested a class of chemical compounds against certain subunits of NMDA receptors and discovered that small changes to the chemical structure of these compounds could change the action of the receptors. For example, one compound called EU1794-4 affected synaptic and extrasynaptic NMDA receptors differently. In particular, this compound activated the receptor at low concentrations of glutamate but inhibited it at high glutamate concentrations. This means that EU1794-4 affects extrasynaptic receptors (which experience low levels of glutamate) and synaptic receptors (which experience high levels of glutamate) in different ways (Figure 1). Thus, in addition to subunit preference, this class of modulator has the potential to target NMDA receptors in certain regions and can change their activity without fully blocking the response. This makes EU1794-4 a unique tool for controlling the neuronal network without causing too many side effects.

The advances reported by Perszyk et al. are numerous. First, we now have the opportunity to specifically study synaptic and extrasynaptic NMDA receptors, especially their subunits – a challenging endeavor nevertheless. Second, Perszyk et al. were able to tailor the actions of these compounds – switching between negative and positive modulation, solely by making subtle changes in the compound structure. Drugs that are customizable for effect – with a specific subunit and regional affinity – could selectively target numerous diseases (Figure 1). Indeed, with a Swiss army knife modulator, a rational treatment of diseases associated with faulty NMDA receptor activity may finally be on the horizon.
